# Identification of Anxiolytic Potential of Niranthin: In-vivo and Computational Investigations

**DOI:** 10.1007/s13659-020-00284-8

**Published:** 2020-11-11

**Authors:** Atul R. Chopade, Prakash M. Somade, Pratik P. Somade, Suraj N. Mali

**Affiliations:** 1 Department of Pharmacology, Rajarambapu College of Pharmacy, Kasegaon, Sangli, Maharashtra 415404 India; 2 Dept. of Physiology, Krishna Institute of Medical Sciences, Karad, Maharashtra India; 3 Government College of Pharmacy, Karad, Maharashtra India

**Keywords:** Pharmacology, Phytoconstituents, Niranthin, Lignan, Anxiolytic activity, EPM models

## Abstract

Anxiety is an unpleasant state, which can critically decrease the quality of life is often accompanied by nervous behaviour and rumination. Niranthin is a lignan isolated from various *Phyllanthus* sources. The literature survey on niranthin highlights wide ranges of the therapeutic potentials. In a present study, based on our previous investigations, we evaluated pure, isolated and characterized niranthin as an anxiolytic agent. The niranthin [6-[(2*R*,3*R*)-3-[(3,4-dimethoxyphenyl)methyl]-4-methoxy-2-(methoxymethyl)butyl]-4-methoxy-1,3-benzodioxole] was purchased from commercial source and further subjected for assessment of its anxiolytic potentials using popular animal models including Elevated plus-maze model/test (EPM) and Light & Dark Exploration test (L&D). GABA-A receptor mediation was evaluated by pretreating the mice with the GABA-A receptor antagonist Flumazenil before the EPM task. Molecular docking simulation studies (pdb id: 4COF) carried out by *Vlife QSAR* software showed that niranthin (docking score: − 62.1714 kcal/mol) have shown comparatively best docking score compared to the standard drug Diazepam (docking score: − 63.1568 kcal/mol). To conclude, Niranthin has probable potential in the management of anxiety disorder. Our in-silico and in-vivo analysis (indirectly) indicated the plausible role of GABA mediation for anxiolytic activity. Although, these studies are preliminary, future in depth experimental explorations will be required to use Niranthin as anti-anxiety drug in near future.

**Electronic supplementary material:**

The online version of this article (10.1007/s13659-020-00284-8) contains supplementary material, which is available to authorized users.

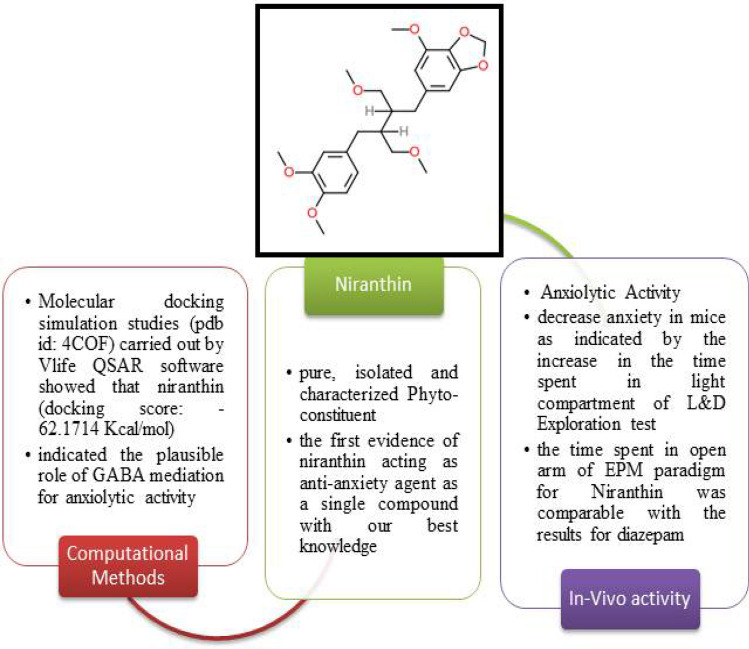

## Introduction

Anxiety, which has neurobiological, cognitive, and behavioral aspects, is one of the leading mental disorders of modern world experienced by children and adolescents [1–4]. It has been also noted that in certain conditions, stress and anxiety might find helpful as they will motivate individuals, but when it becomes excessive, it leads disturbances in psychological states of individuals. Anxiety is a central nervous system (CNS) disorder with negative emotional state, causing uneasiness, fear, etc. in response to factors perceived from internal or external sources [1–6]. It has also proved that incidence of morbidity associated with anxiety associated community found to be very high [7–10]. In a very recent report, authors mentioned that the neurobiology of anxiety is still unknown [11]. However, like other CNS disorders, is also linked with CNS neurotransmitter imbalances. There are four neurotransmitters playing key roles in mood regulations, which are norepinephrine, gamma-aminobutyric acid (GABA), serotonin, and dopamine. Glutamate is found to be most occurring neurotransmitter and found to be assisted by three catecholamines. GABA, glycine, and serotonin are considered as inhibitory neurotransmitters. Gamma-aminobutyric acid is found to be assisting neurons to recover after impulse transmission and thereby reducing the stress and anxiety. GABA also regulates both epinephrine and norepinephrine in order to reduce neuronal excitability during neuronal transmission.

Pharmacotherapy for the management of anxiety disorders includes varieties of drugs such as tricyclic antidepressants (TCA), monoamine oxidase inhibitors (MAOI’s), and serotonin reuptake inhibitors (SSRI’s). These medications ultimately lead alterations in neuronal chemistry via amplification and regulations of NTs [2].

Our conventional pharmacotheraphy for anxiety management has lots of adverse side effects which includes but not limited to sexual dysfunction, dependence liability and psychomotor imbalances [12]. There is an urgent need for identifications of phytoconstituents, which might be developed into safe, effective, and cost-effective anxiolytic agents in near future. A number of research studies were carried out on anti-anxiety activity of various medicinal plants reporting anxiolytic activity of plant in forms of standardized extracts, but there are only few evidences reporting anxiolytic activity of pure isolated, characterized compounds in plant. There are many people throughout the world, who would opt to use complementary and alternative medicines for treatment of their psychiatric symptoms [13, 14]. Majority portion of psychiatric patients believe that these medicines are having lesser side effects (which is not the case for every time) and also available at very cheap prices [12]. Furthermore, it has also been evident that traditional medicines are still part of our culture and customs, especially involving African communities [14].

The lignan family of natural products includes compounds with important and wide range of medicinal properties [15]. There are many reports on wide bioactivity of various Phyllanthus species having anti-inflammatory, anti-viral, antimicrobial, anti-cancer and anxiolytic potentials [16–19]. Niranthin, a lignin, isolated from the the plant *Phyllanthus amarus,* also shown to have anti-inflammatory, anti-allodynic properties, anti-viral and cytotoxic effects (K-562 cell line) [16–19]. Chowdhury et al., have shown niranthin capability as a potent anti-leishmanial agent [20]. Recently Conrado et al. 2020 have reaffirmed anti-leishmanial and anti-trypanosomal Activity activity of niranthin and other lignans from Niranthin [21]. Recently, our analysis for *P.amarus* and *P. fraternus* standardized extracts showed antxiolytic potential in mice models. In order to shade more lights on these activities, we carried out analysis with single, isolated niranthin molecule [22–24].

Henceforth, our present study has been focused to carry out anti-anxiety activity of pure, isolated and characterized niranthin in various animal models in-vivo as assessed by light/dark box, EPMT and motor coordination test [25–28]. We have also carried out in-silico molecular modelling studies in order to support our current findings [22, 24, 29–36]. Molecular docking simulations used in our current study will also shade more lights on GABA assisted/mediated role of niranthin as anti-anxiety agent.

## Material and Methods

### Animals Used

Swiss albino male mice of either sex were used in the present study. Mice weighing approximately 25–30 g were utilized. Mice were grouped as 6/cage and housed in the standard laboratory conditions of light (12 h each of dark and light cycles) and temperature. Food and water were provided ad libitum. The experiments were carried out during the time frame of 9.00 am to 3.00 pm. Animals were fasted (of food but not water) for 12 h before the set of experimental trials. The acclimatization of mice to the lab environment was ensured (i.e., housed for at least 10 days prior to first set of trials). The experimental protocols were developed as per the ethical principles/guidelines and are approved by institutional animal ethical committee (constituted for the purpose of control and supervision of experimental animals by ministry of Environmental and Forests, Government of India, New Delhi) and were followed during the conduct per the guidance stated above. The approved protocol numbers were RCP/18 19/P-20.

### Chemical Agents

The Niranthin [6-[(2*R*,3*R*)-3-[(3,4-dimethoxyphenyl)methyl]-4-methoxy-2-(methoxymethyl)butyl]-4-methoxy-1,3-benzodioxole] purchased (Product code: N006, Lot. no.: T18C277) from Natural Remedies Pvt. Ltd., Bangalore. Purity of Niranthin was determined by the manufacturer by HPLC area normalization and was certified above 95.0%. Dimethylsulphoxide (DMSO), sodium chloride (NaCl), all from Loba chemicals) were used in this investigation.

### Evaluation of Anxiolytic Potential using Elevated Plus Maze Test (EPM)

In the present study, the elevated plus maze (EPM) apparatus as described by Pellow et al. [25] and for mice as specified by Lister et al. [26] was utilized to assess anxiolytic potential of P. amarus extracts. We have used the popular EPM test for the assessments of behavioral aspects for anxiety. When we placed rodents on the EPM, due to fear to height they were subjected to anxiety. The manifestations to anxiety can easily be accessed by looking at rodents to stay at safer places and with decremented motor activity. Typical, EPM apparatus accompanies the two open arms (37 × 5 cm) and two enclosed arms (37 × 5 × 12 cm) with 12 cm high wall arranged. This arrangement makes sure that same types of arms will be opposite to each other. A central square (5 × 5 cm) was connected to arms. We have kept the wooden apparatus to a 25 cm height above the floor. All mice were allowed to place separately in EPM center in such a way that they faced the open arm. For a period of 5 min each, we recorded the time spent in open and enclosed arm. We have ultilized each animal only single time and furthermore, we conducted test protocol during specific time of day as mentioned in Sect. 2.1. A simple rational behind this is that the open arms are more fear-provoking and that the ratio of the time spent on open, closed arms or entries into open-closed arms reflect the relative “safety” of closed arms compared with the relative “fearfulness” of open arms. It has been believed that anxiolytics will cause increment into and time spent on open arms of EPM apparatus. For cleansing of the EPM apparatus, we utilized hydrogen peroxide.

### Light and Dark Exploration Test (L & D)

Light and Dark Exploration (L & D E) test method represents the natural habit of animals like the dark place, i.e., they tend to avoid entry into and reduce spontaneous exploratory behavior in the brightly illuminated area; a natural tendency when a rat/mice is exposed to an unfamiliar environment. During a 5-min period, animals were permitted to freely investigate a new atmosphere comprised of two different compartments: protected (dark) and unprotected (light). Anxiolytic compounds change the natural habit of animals to light and increase the time spent in the light compartment. The dark and other bright are the two compartments boxes of the L&D apparatus. As there will be reduction of aversion to light compartments by anxiolytics, rodents will spend more time in that compartment. As opposite, to above fact, the agents causing the anxiety, will force rodents to spend more time in the dark compartment. This apparatus is made of wood and having dimensions of 45X27X27 cm. This box was allowed to open and illuminated with incandescent lamps, 65 lx. We have recorded for the duration of 5 min, the number of crossings and time spent in L & D compartments, after individually placing naïve mice in the center of the L compartment [22, 24].

### Assessment of Motor Activity

It has been well reported that barbiturates, benzodiazepines like compounds may result into the impairment of Rota-rod apparatus. For evaluation of effects on motor coordination, we utilized the Rota-rod apparatus. This Rota Rod apparatus, model—K19616-2 Inco, Ambala has a bar and is subdivided into three compartments by discs. We allowed the rotation of the bar at the speed of 22 rpm. Those mice, which did not remain on the bar for two consecutive periods of 150–200 s, were excluded, eliminated before 24 h of experiment. After proper selection we have intraperitoneally administered the drugs, 30 min before conducting the test. Results were representing the time for which animals were supposed to stay on the Rota-rod (Cut off time = 150 S).

### Assessment of Involvement of GABA Receptor

In order to evaluate the involvement of the GABA receptor for anxiolytic effects shown by Niranthin; we used popular GABA-benzodiazepine antagonist, Flumazenil (FLU). Flumazenil (FLU) at a dose of 2.5 mg/kg was given along with Niranthin groups and Diazepam (DZP) to evaluate the effects utilizing EPM model as per procedures given above (Sects. 2.3 and 2.4).

### Docking Methodology and In-silico Analysis

#### Ligand Preparation

We allowed converting 2D structures into 3D structures by utilizing the popular sketching Chem Draw Ultra 8.0. These 3D structures were then constructed to energy minimization process by using batch optimization for a set of molecules. The MMFF is used for molecular mechanics. The systematic search method was utilized for generation of the conformers. We have ranked the docking results according to the decrements in docking energies of the different possible conformers for each of the ligands [22, 24].

#### Preparation of Target Protein

Molecular docking studies were performed using Vlife MDS 4.6.1 version. In recent studies, varied targets were screened for biological activities, which formed the basis for present molecular screening methodology [22, 24, 29–36]. 3D X-ray crystallographic structure of the GABA-(A) homopentamer receptor (PDB Code: 4COF) was retrieved from the Protein Data Bank (www.rcsb.com) [37]. The receptor was extracted by X-ray diffraction method at a resolution of 2.97 A. Benzamidine, a novel agonist, is co-crystallized with GABAA R. GABAA R-β3cryst has a closed β9-β10 loop, being in an agonist bound state, but the pore was shut, consistent with a desensitized conformation. GABAAR-β3cryst was in agreement with our electrophysiological recordings of benzamidine induced desensitising currents measured in HEK cells at saturating concentrations (10 mM), which were used in crystallisation (33 mM). Furthermore, in heteromeric GABAARs, swapping the β-subunit intracellular border with the equivalent nAChR residues ablates desensitization. Thus, by using Vlife MDS the protein molecules was reconstructed by addition of hydrogen in protein. The protein molecules were saved into the.mole2 format and used for further processing.

### In-silico Analysis

We have evaluated the in-silico predictions of Molecular Properties and Drug-likeness of Niranthin for predictions of passive intestinal absorption and brain penetration, as a function of lipophilicity and apparent polarity using popular web based tool called Web Molecular Editor v1.5.1 (https://www.molsoft.com/mprop/) [29–36].

### Statistical Analysis

A Graph pad Prism software version 6.01©, 1992–2012 was used by us for the statistical calculation. We represented all data in terms of mean ± SEM (standard error of the mean). Statistical significance (P-value < 0.001) was obtained with control. Furthermore, we have also carried out one way ANOVA (one-way analysis of variance) for statistical analysis and treatment with Dunnett’s multiple comparison test.

## Results

### Effects of Niranthin on the EPM Model

**Fig. 1:**
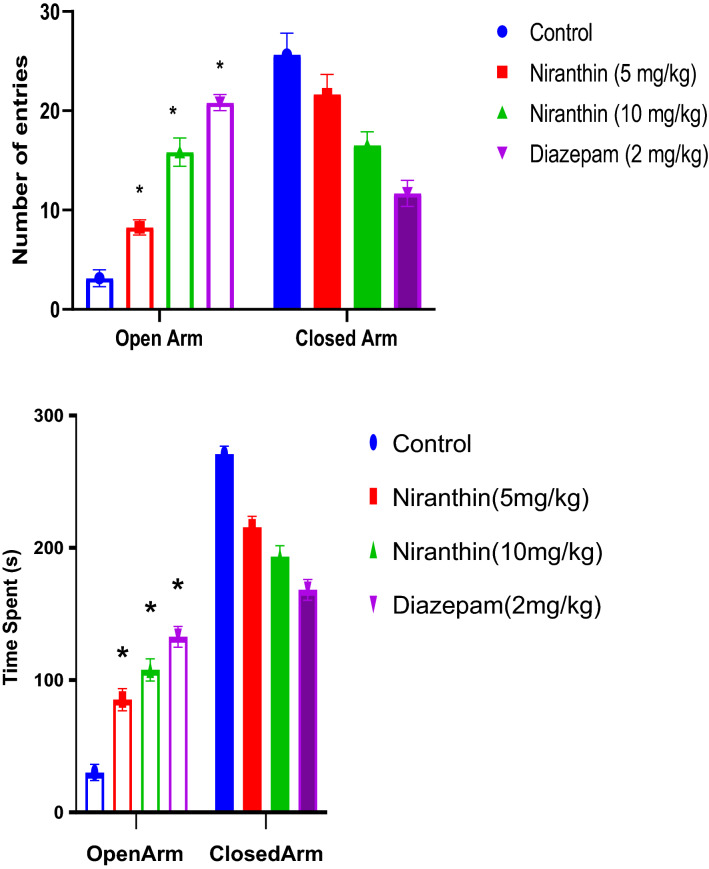
**a** Effect of Niranthin on number of entries in the EPM model. Value in figure is expressed as Mean ± SEM (standard error of mean), where, *P < 0.001 significant. **b** Effect of Niranthin on time spent in the EPM model. Value in figure is expressed as Mean ± SEM (standard error of mean), where, *P < 0.001 significant

The number of entries in open and closed arms of the EPM apparatus after drug treatment in Control, Niranthin and Diazepam 2 mg/kg treated animals is presented in the (Fig. 1a). The Niranthin demonstrated anxiolytic potential in mice as indicated by the increase in number of entries in open arm of EPM paradigm compared with control group (p < 0.001). Also, the number of entries reported in open arm of EPM paradigm for Niranthin was comparable with the number of entries observed for diazepam. The time spent in an open and closed arms of the EPM apparatus after drug treatment in Control, Niranthin (5 and 10 mg/kg), and Diazepam 2 mg/kg treated animals are presented in the (Fig. 1b). The Niranthin demonstrated anxiolytic potential in mice as indicated by the increase in time spent in open arm of the EPM paradigm compared with control group (p < 0.001). Also, the time spent in open arm of EPM paradigm for Niranthin was comparable with the results for diazepam.

### Effects of Niranthin on the L & D Exploration Test

**Fig. 2:**
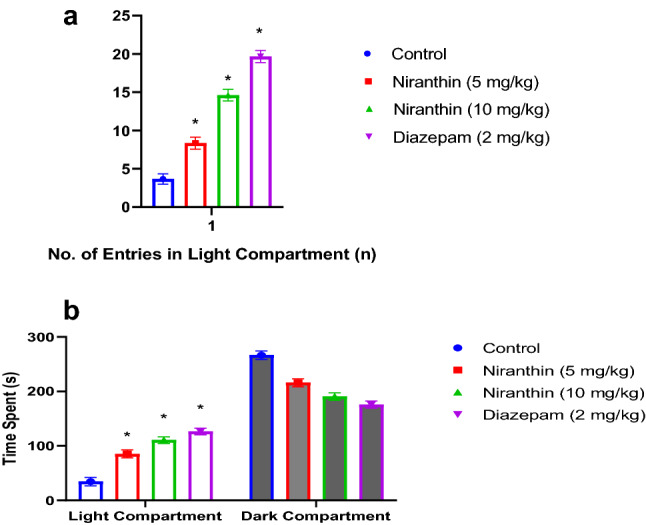
**a** Effect of Niranthin on number of entries in the light compartment for L & D exploration test. Value in figure is expressed as Mean ± SEM (standard error of mean), where, *P < 0.001 significant. **b** Effect of Niranthin on time spent in the L & D exploration test. Value in figure is expressed as Mean ± SEM (standard error of mean), where, *P < 0.001 significant

The number of entries in the light compartment of L & D exploration test after drug treatment in Control, Niranthin and Diazepam treated animals were presented in the (Fig. 2a). The Niranthin demonstrated anxiolytic potential in mice as indicated by the increase in number of entries in the light compartment of L & D exploration test compared with control group (p < 0.001). For Niranthin treated mice, the number of entries in light compartment of L&D E test apparatus was comparable with the results for diazepam treated mice. The time spent in the dark and light compartments of L & D exploration test after drug treatment in Control, Niranthin and Diazepam treated animals were as per (Fig. 2b).

The Niranthin decreased anxiety in mice as indicated by the increase in the time spent in light compartment of L&D Exploration test compared with normal saline treated animals (p < 0.001). Also, the time spent in light compartment was comparable with the results for diazepam.

### Effects of Niranthin on the Motor Activity

**Fig. 3:**
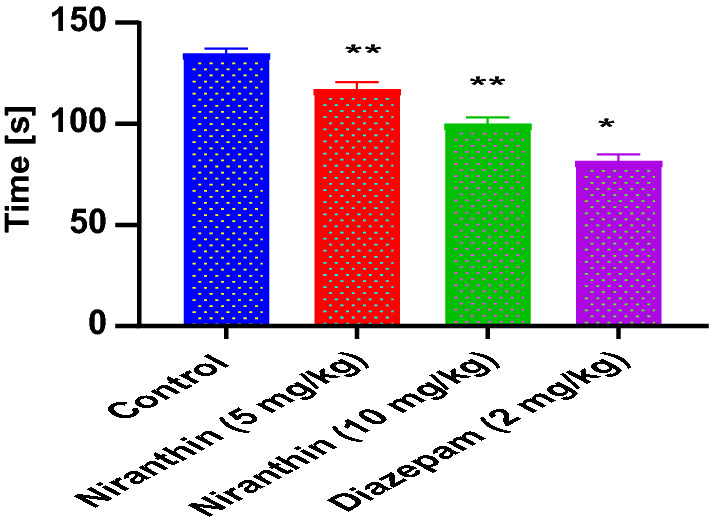
Effect of Niranthin on locomotor activity. Value in figure is expressed as Mean ± SEM (standard error of mean), where, *P < 0.001 and **P < 0.01 significant

The score of motor activity after drug treatment in Control, Niranthin and Diazepam treated animals were as per (Fig. 3). The results for Niranthin 5 mg/kg treated animals did not show significant change in locomotor activity compared with the results for control group. Also, the results for Niranthin 10 mg/kg treated animals were comparable with the results for diazepam treated mice.

### Blockade of the Anxiolytic Effect of Niranthin by Flumazenil

**Fig. 4:**
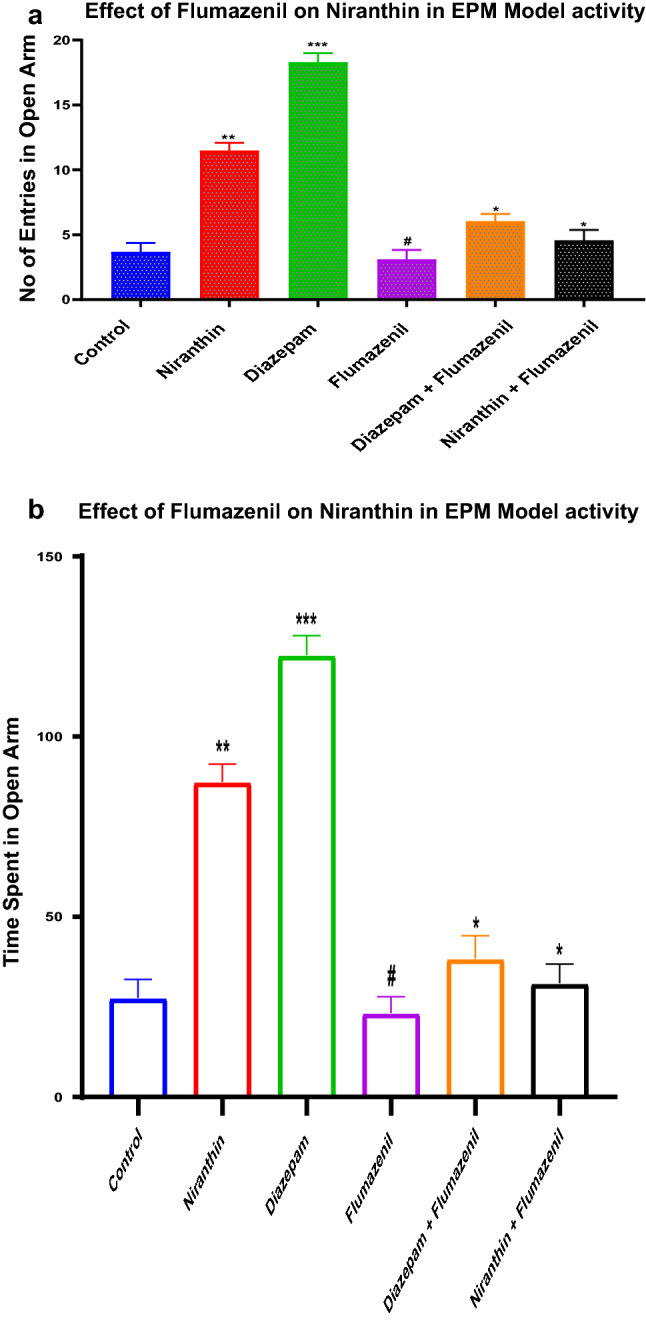
**a** Elevated Plus Maze task effects of Flumazenil on Niranthin pretreament. Values are in mean ± SEM (n = 6): **denotes p < 0.01as compared to control group of young mice. (One-way ANOVA followed by Dunnett’s test). **b** Elevated Plus Maze task effects of Flumazenil on Niranthin pretreament. Values are in mean ± SEM (n = 6): ** denotes p < 0.01as compared to control group of young mice. (One-way ANOVA followed by Dunnett’s test)

Both in the EPM test and L & D Exploration test, Flumazenil reversed the effect of diazepam and Niranthin (at studied doses of 5 and 10 mg/kg) on the number of and time spent in the open arm of EPM apparatus and light compartment of L&D Exploration apparatus, suggestive of possible mechanism of action of Niranthin via GABA_A_ receptor. The summarized results of EPM test and L & D Exploration test are shown in (Fig. 4a, b) respectively.

### Docking Predictions

**Fig. 5:**
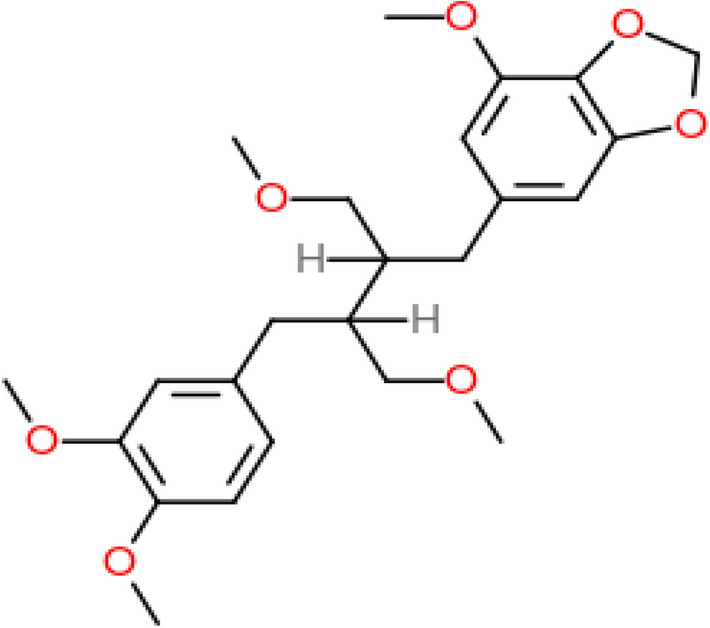
Molecular structure of Niranthin

**Fig. 6:**
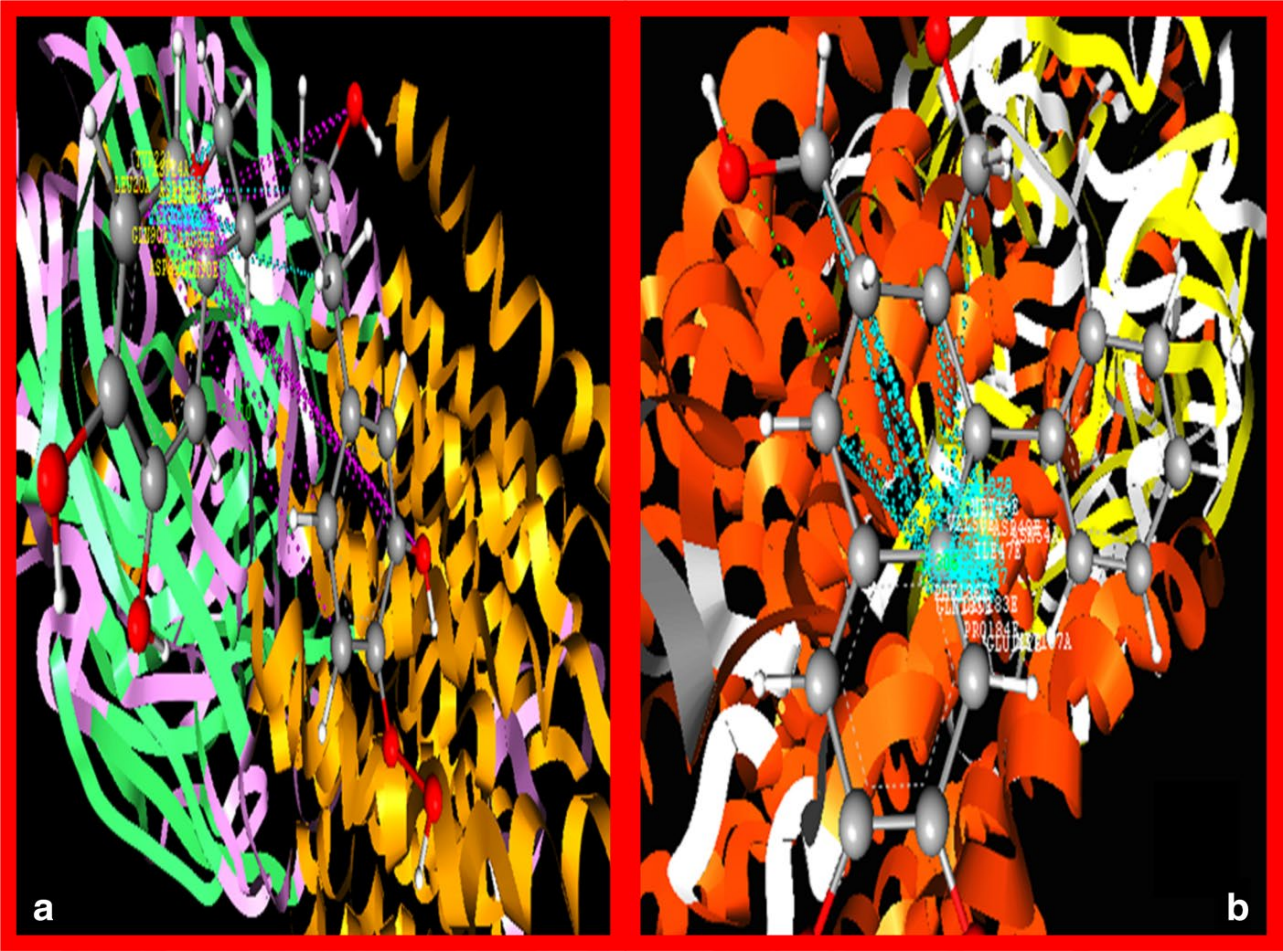
Molecular Docking interactions of Diazepam (**a**) and Niranthin (**b**) with Crystal structure of a human gamma-aminobutyric acid receptor, the GABA (A) R-beta3 homopentamer receptor (PDB Code: 4COF)

**Fig. 7:**
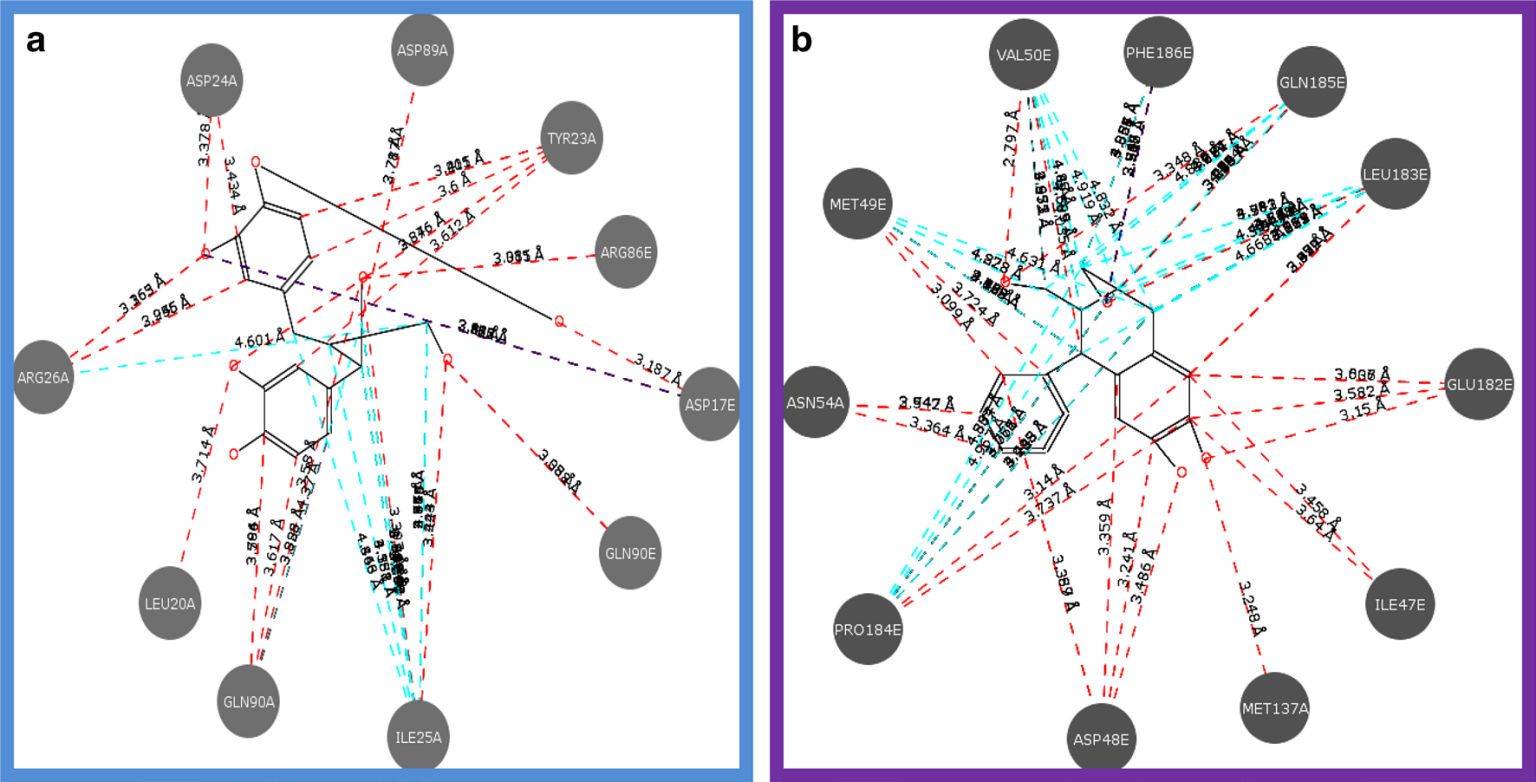
Two dimensional Molecular interactions of Diazepam (**a**) and Niranthin (**b**) with Crystal structure of a human gamma-aminobutyric acid receptor, the GABA (**a**) R-beta3 homopentamer receptor (PDB Code: 4COF)

In the current study, the molecular docking study was performed by using Vlife QSAR software. The software uses BioPredict tools to perform the GRIP docking. Molecular docking analysis against receptor contains crystal structure of a human gamma-aminobutyric acid receptor, the GABA (A) R-beta3 homopentamer injuries to the extent of 47–70% whereas; the co-administration of benzamidine prevented it significantly. Molecular structure of Niranthin is shown in (Fig. 5). It was observed that Niranthin (docking score: − 62.1714 kcal/mol) have shown best docking score compared to the standard drug Diazepam (docking score: − 63.1568 kcal/mol). (Fig. 6) depicts the 3D Interaction poses of Niranthin and Diazepam on protein (pdb id: 4COF). Additionally, the 2D Interactions of Niranthin and Diazepam molecules against the active site of GABA (A) Rbeta3 homopentamer receptor are shown in (Figs. 6, 7).

### In-Silico Analysis of Niranthin

**Fig. 8:**
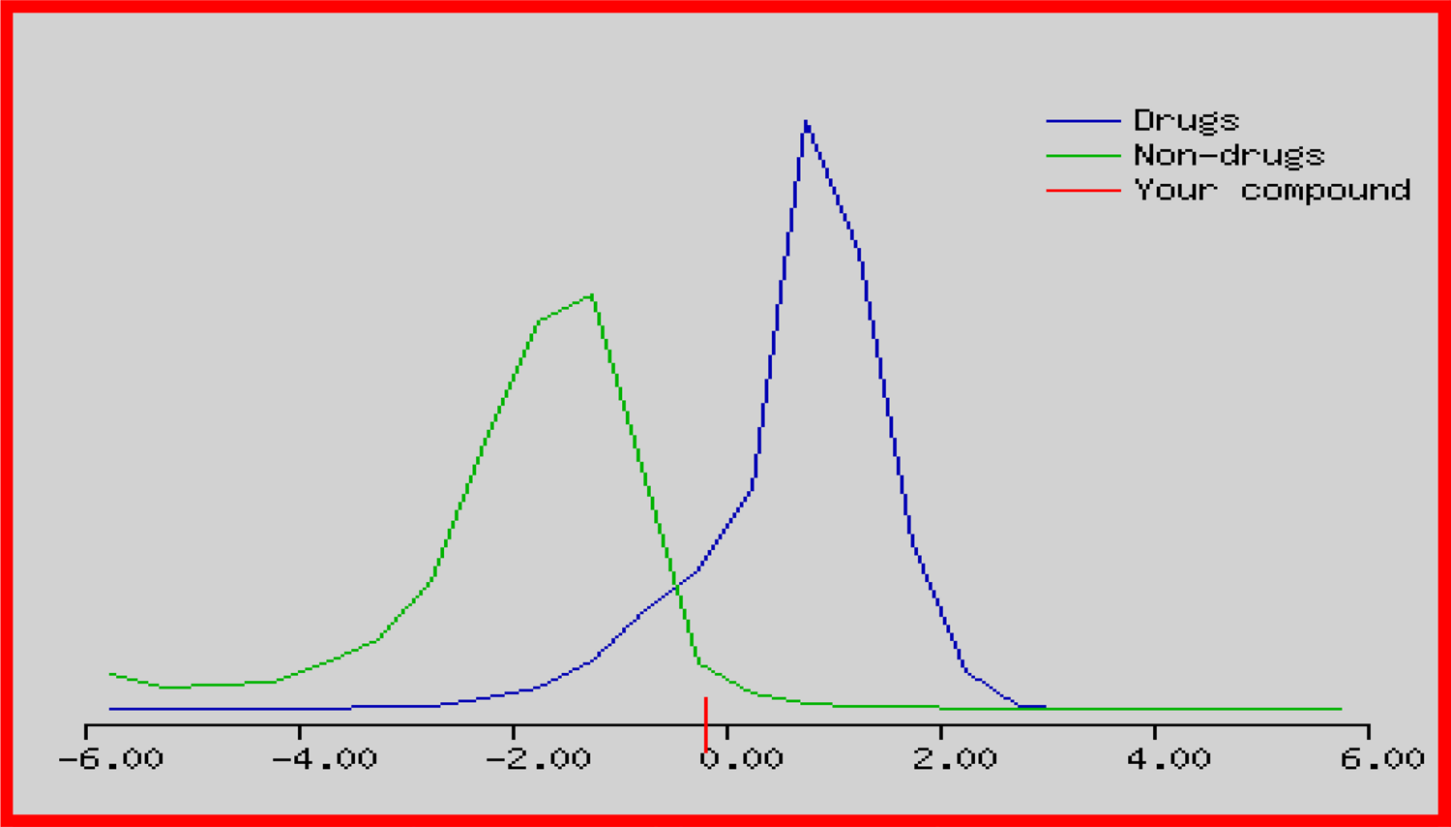
Drug-likeness model score plot of Niranthin for its antianxiety potential

In-silico studies were performed for predictions of Molecular Properties and Drug-likeness of Niranthin by Web Molecular Editor v1.5.1 (https://www.molsoft.com/mprop/). It predicts an overall drug-likeness score using and Molsoft's chemical fingerprints. Drug-likeness model score plot of Niranthin for its antianxiety potential is depicted in (Fig. 8). The summarized details of in-silico predictions of Molecular Properties and Drug-likeness of Niranthin are given in (Table 1).

**Table 1:** In Silico predictions of molecular properties and drug-likeness of Niranthin

Properties	Molecular properties of Niranthin
Molecular formula:	C_24_ H_32_ O_7_
Molecular weight:	432.214803 g/mol
Number of HBA (Hydrogen bond acceptor)	7
Number of HBD (Hydrogen bond donor)	0
MolLogP (Octanol/water partition coefficient)	3.68
MolLogS (water solubility Log(Mol/L))	− 3.61 (in Log(moles/L)) 105.94 (in mg/L)
MolPSA (molecular polar surface area (PSA) and volume)	56.51 A^2^
Molecular volume	435.67 A^3^
pKa of most basic/acidic group	< 0./18.11
Number of stereo centers	2
BBB score: the blood–brain barrier (BBB) score: 6-high,0-low (https://doi.org/10.1021/Acs.Jmedchem.9b01220*)*	3.99 s

## Discussion

**Fig. 9:**
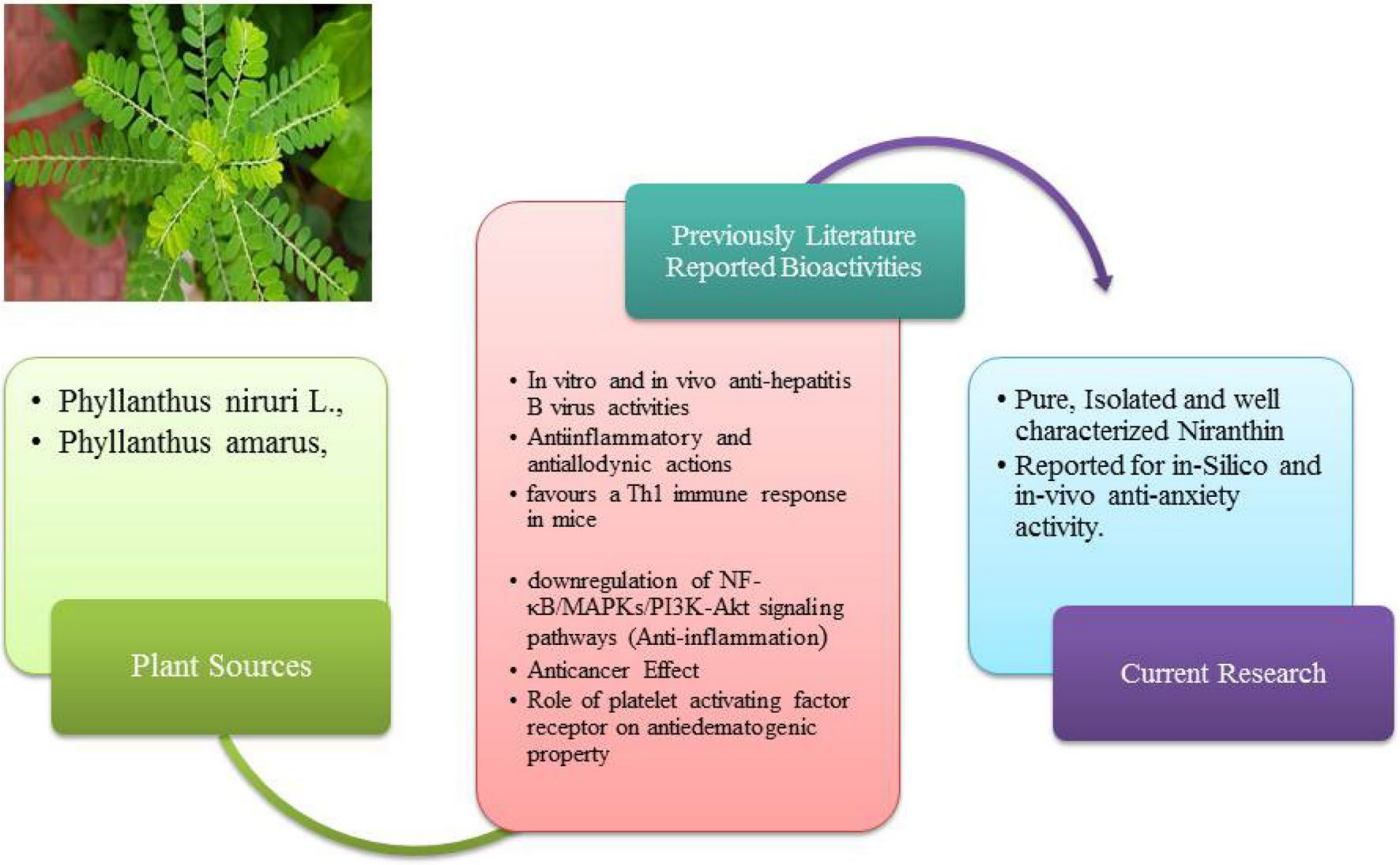
previously literature reported pharmacological potential of isolated niranthin and current study

Figure 9 illustrates previously reported pharmacological potential of isolated niranthin along with previous sources [38]. In order to evaluate the anxiolytic potential of Niranthin, we have studied three behavioral animal models of anxiety; EPM apparatus, L& D E test, and LM activity. As they minimize confounding factors of other conditioned assays and produce reproducible paradigm for creation of anxiety in normal rodents, henceforth we utilized the aforementioned models. In this way, we would be able to screen central nervous system actions giving more details about anxiety and psychomotor performance. For better search of new benzodiazepine-like anxiolytic agents, we utilized the well-reported EPM test protocol. EPM is based on the behavioral aspects of animals, when exposed to an elevated maze alley, which henceforth provide an approach-avoidance conflict. Animals were observed to spend more time in the closed arms as compared to placing of open arms. When an animal spends more time in open arms it is assumed that it is in good mood and free from anxiety. In this study, Niranthin (5 and 10 mg/kg), separately, produced significant effect in a dose dependent manner compared to Diazepam group. Also, results of 5 mg/kg groups showed that the anxiolytic activity was without any impairment in motor activity.

Anxiolytics tend to increase the number of entries; time spent, and rears in the bright arena of L & D E test apparatus. However, in this model, compared to anxiety control, three extracts of the Niranthin in two gradual doses (5 and 10 mg/kg), separately, produced significant effect in a dose dependent manner compared to Diazepam group, i.e. increase the time spent on the animals in the light box. It was noted that the reduction of spontaneous motor activity could be related to the calmness/sedative effect. In the present study, the motor activity results demonstrated dose-dependent sedative activity of Niranthin. The effect of Niranthin was antagonized by flumazenil, indicating that niranthin -induced anxiolytic effect was mediated by γ-aminobutyric acid (GABA)ergic transmission via benzodiazepine (BDZ)-responsive GABA-A receptors [38]. Thus, present study preliminarily indicated that Niranthin produced anti-anxiety property through modulation of GABA-Aergic transmission [39]. Furthermore, in future, these results could be explored with more advanced patch-clamp method for additional verifications.

The results obtained in our study would suggest the probable anxiolytic potential of Niranthin. The molecular docking studies have revealed that these effects of the Niranthin could be due to the interaction with the GABA/benzodiazepine receptor complex in the brain. It is noteworthy to mention that Niranthin was observed as deeply embedded in the allosteric site surrounded by highly electronegative residues and forms hydrogen bonding; having docking score of − 62.1714 kcal/mol. It demonstrates that Niranthin possesses most prominent activity against GABA receptors. In-silico models clearly demonstrated the passing of Niranthin, via BBB barrier. From our current study, a possible positive modulation of the GABA-_A_/benzodiazepine receptor complex in relieving anxiety has been noted as we tested and recorded the effects of the Niranthin using selective blocker as well as parallel in-silico analysis.

However our current in-vivo study suggests that at the studied doses Niranthin possess significant anxiolytic activity and could be successfully used for the treatment of anxiety after careful optimization of dosage in the future. A further in-depth study utilizing patch clamp recording technique of gamma-aminobutyric acid receptors by inducing macroscopic chloride ionic currents would add clear insights in the proposed mechanism of Niranthin in present study [40]. Hence, further investigations are deemed necessary for elucidating the exact mechanism and detailed investigation of efficacy of Niranthin.

## Conclusion

Our current study clearly indicates the pure Phytoconstituent niranthin has suggestive indirect potential in the management of anxiety disorder. The molecular docking results indicated the plausible role of GABA- mediation for anxiolytic activity. Furthermore, in-depth study utilizing patch clamp recording technique of gamma-aminobutyric acid receptors by inducing macroscopic chloride ionic currents might add direct clear insights in the proposed mechanism of Niranthin, in future. Although, our in-silico and in-vivo studies are preliminary (indirectly suggesting mechanisms), future in depth experimental explorations such as patch clamp method will be required to use as anti-anxiety drug in near future.

## Electronic supplementary material

Below is the link to the electronic supplementary material.

Supplementary file1 (PDF 509 kb)
