# Supplementary material for: Identification of Anxiolytic Potential of Niranthin: In-vivo and Computational Investigations
Source: Nat Prod Bioprospect. 2020 Nov 11;11(2):223–33. doi: 10.1007/s13659-020-00284-8 (PMC7981351; doi:10.1007/s13659-020-00284-8)
Supplement: Supplementary file 1 — Supplementary file1 (PDF 509 kb) [file 13659_2020_284_MOESM1_ESM.pdf]

Supplementary Information

The observed ^{1}H-NMR differences data of these lignans are coherent with the literature data, as well as their 13C-NMR data.

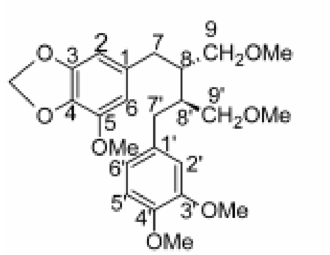

Niranthin (C)

Niranthin: ^{1}H NMR[1,2] (500 MHz, CDCl3), δ: 6.30 (1H, *d*, *J*=1.15 Hz, H-2), 5.93 (2H, *s*, -OCH2O-), 3.83 (3H, *s*, 5-OCH3), 6.25 (1H, *d*, *J*=1.15 Hz. H-6), 2.59 (2H, *t*, *J*=5.15, H-7), 2.63 (2H, *t*, *J*=8.05, H-7), 2.02 (1H, *m*, H-8), 3.30 (2H, *s*, H-9), 3.30 (3H, *s*, 9-OCH3), 6.62 (1H, *d*, *J*=1.70 Hz, H-2'), 3.86 (3H, *s*, 3'-OCH3), 3.82 (3H, *s*, 4'-OCH3), 6.76 (1H, *d*, *J*=8.00 Hz, H-5'), 6.65 (1H, *dd*, *J*=2.30 Hz, 8.05 Hz, H-6'), 2.66 (2H, *dd*, *J*=2.85 Hz, 6.85 Hz, H-7'), 2.67 (2H, *t*, *J*=6.90 Hz, H-7'), 2.02 (1H, *m*, H-8'), 3.30 (2H, *s*, H-9'), 3.30 (3H, *s*, 9'-OCH3).

^{13}C NMR (125 MHz, CDCl3), δ: 135.7 (C-1), 103.2 (C-2), 148.6 (C-3), 133.6 (C-4), 101.3 (-OCH2O-), 143.4 (C-5) 56.5 (5-OCH3), 108.0 (C-6), 35.5(C-7), 40.9 (C-8), 72.6 (C-9), 59.0 (9-OCH3O), 133.2 (C-1'), 112.1 (C-2'), 147.1 (C-3'), 55.9 (3'-OCH3), 148.7 (C-4'), 55.8 (4'-OCH3), 110.9 (C-5'), 121.1 (C-6'), 35.0 (C-7'), 40.8 (C-8'), 72.5 (C-9'), 58.9 (9'-OCH3).

References:

[1] Noor, N.A.M., Nafiah, M.A., Tuan Johari, S.A.T., Hasnan, M.H.H., Tan, S.P., Liew, S.Y. and Supratman, U., 2019. Anticancer Effect of Hypophyllanthin, Niranthin and Lintetralin From Phyllanthus amarus on HeLa Cells And NIH/3T3 Cells. *International Journal of Recent Technology and Engineering*, *8*(2S7), pp.106-110.

[2]Manju Singh , Neerja Tiwari , Karuna Shanker , Ram Kishore Verma , Anil Kumar Gupta & Madan Mohan Gupta (2009) Two new lignans from Phyllanthus amarus , Journal of Asian Natural Products Research, 11:6, 562-568, DOI: 10.1080/10286020902939174

CERTIFICATE OF ANALYSIS

Date: 23.10.2019

NIRANTHIN

Product code : **N006**

Lot. no. : T18C277

Storage : At 18-25°C

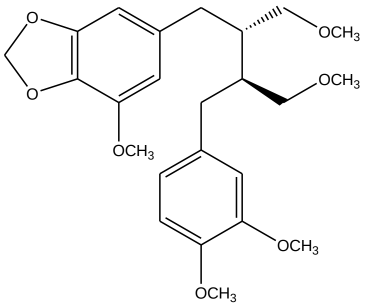

Molecular formula : $C_{24}H_{32}O_7$

Formula weight : 432.51

CAS No : 50656-77-4

Valid up to : March 2022

TESTS		RESULTS
**Description**	:	Off-white powder
**Solubility**	:	Soluble in chloroform. Insoluble in water

Identity & Purity (by Spectroscopy & Chromatography)

**TLC & HPTLC**	:	Gives a single principle spot
**UV absorption**	:	Exhibits maxima 233& 279 nm
**FTIR**	:	Characteristic of ' Niranthin'
**^{1}H and ^{13}C NMR**	:	Characteristic of 'Niranthin '
**MASS**	:	Characteristic of ' Niranthin'
**HPLC purity (By area normalization)**	:	95.0%

ANALYST

AUTHORISED SIGNATORY

HPLC-CONDITIONS

Chromatographic system: Shimadzu High Performance Liquid Chromatographic System LC 2010CHT with UV detector in combination with Class LC solution software.

Chromatographic conditions

Column	:	Pinnacle DB C18, 5µ (250 x 4.6 mm)		
Column oven temperature	:	27 ± 1°C		
Mobile phase	:	**C –** 0.140 g of anhydrous potassium dihydrogen orthophosphate (KH_2PO_4) dissolved in 900 ml of HPLC grade water & add 0.5 ml of orthophosphoric acid and made up to 1000 ml with water. The solution was filtered through 0.45µ membrane filter and degassed in a sonicator for 3 minutes.		
	:	**B-**Acetonitrile (HPLC grade)		
Flow rate	:	1.5 ml		
Detection Wave length	:	230 nm		
Injection volume	:	20.0µl		
Gradient	:	Time	C conc (buffer)	B.Conc (Acetonitrile)
	:	0.01	10	90
	:	10.00	40	60
	:	15.00	80	20
	:	20.00	100	20
	:	22.00	100	0
	:	25.01	10	90
	:	30.00	Controller	Stop
Sample preparation	:	About 10 mg of the sample was accurately weighed to a 10 ml volumetric flask. ~5 ml of methanol was added & sonicated cooled & made up to 10 ml.		

HPLC CHROMATOGRAM

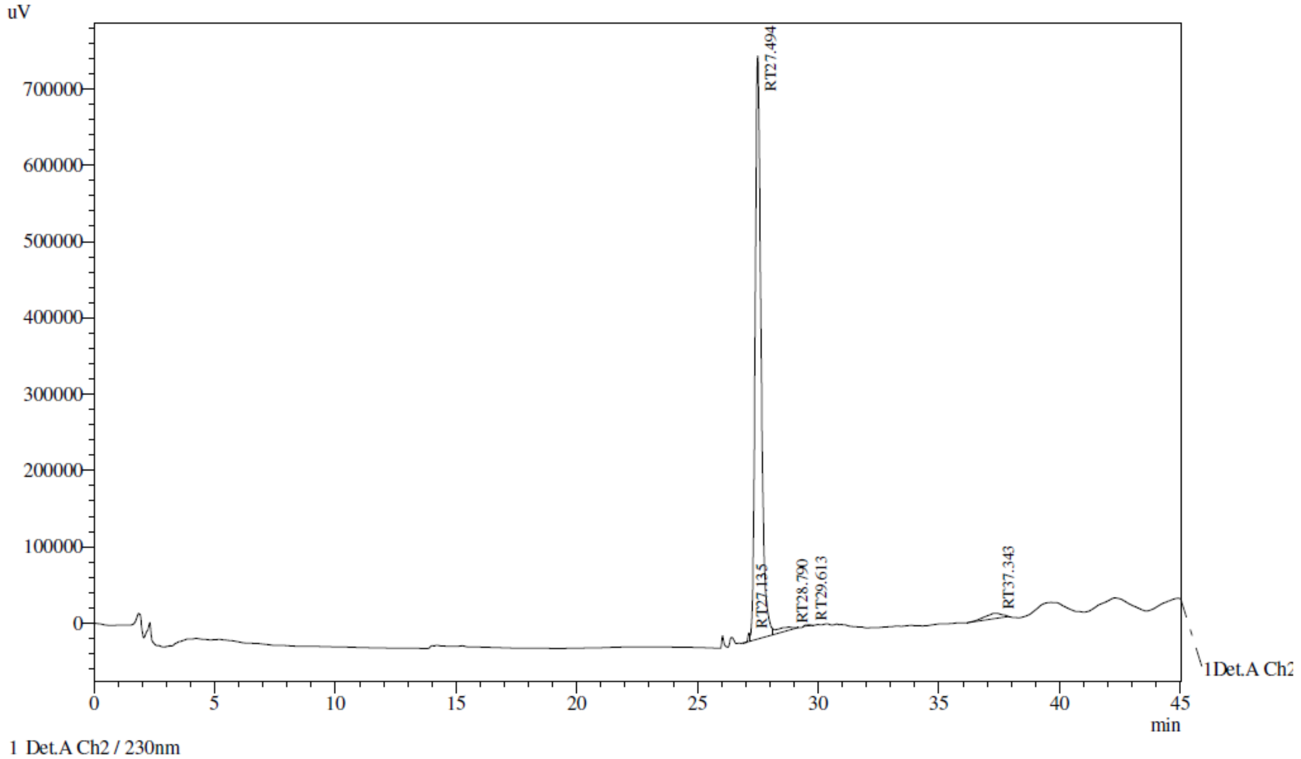

1 Det.A Ch2 / 230nm

PeakTable

Detector A Ch2 230nm

Peak#	Ret. Time	Area	Height	Area %	Height %
1	27.135	55833	11009	0.406	1.398
2	27.494	13086210	763623	95.045	97.003
3	28.790	256553	4220	1.863	0.536
4	29.613	19034	1339	0.138	0.170
5	37.343	350757	7027	2.548	0.893
Total		13768388	787219	100.000	100.000
